# The analysis of oral microbial communities of wild-type and toll-like receptor 2-deficient mice using a 454 GS FLX Titanium pyrosequencer

**DOI:** 10.1186/1471-2180-10-101

**Published:** 2010-04-06

**Authors:** Jongsik Chun, Kap Y Kim, Jae-Hak Lee, Youngnim Choi

**Affiliations:** 1 School of Biological Sciences and Institute of Microbiology, Seoul National University, Seoul, Republic of Korea; 2 Interdisciplinary Program in Bioinformatics, Seoul National University, Seoul, Republic of Korea; 3 Programs in Oromaxillofacial Infection & Immunity and BK21 CLS, Seoul National University and Dental Research Institute, Seoul, Republic of Korea

## Abstract

**Background:**

Although mice have long served as an animal model for periodontitis, information on the composition of their indigenous oral microbiota is limited. The aim of the current study was to characterize mouse oral bacterial flora by applying extensive parallel pyrosequencing using the latest model pyrosequencer, a Roche/454 Genome Sequencer FLX Titanium. In addition, the effect of Toll-like receptor (TLR) 2 deficiency on oral microbiota was evaluated.

**Results:**

Eight oral bacterial communities of wild-type (n = 4) and TLR2 knock-out (n = 4) C57BL/6 mice were characterized by analyzing 80,046 reads of 16S rRNA genes obtained by pyrosequencing. Excluding the PCR primers, the average length of each sequencing product was 443 bp. The average species richness of the murine oral bacterial communities was estimated to be about 200, but the communities were dominated by only two main phyla and several species. Therefore, the bacterial communities were relatively simple. The bacterial composition of the murine oral microbiota was significantly different from that of humans, and the lack of TLR2 had a negligible effect on the murine oral microbiota.

**Conclusion:**

Pyrosequencing using the Roche/454 FLX Titanium successfully characterized mouse oral bacterial communities. The relatively simple oral bacterial communities of mice were not affected by TLR2 deficiency. These findings will provide a basis for future studies on the role of periodontal pathogens in the murine model of periodontitis.

## Background

Mice do not develop periodontitis naturally, but experimental periodontitis can be induced by inoculating mice with a periodontal pathogen such as *Porphyromonas gingivalis *[1]. Experimentally induced periodontitis in mice has served as an animal model for human periodontitis. Since periodontitis is caused by a dental biofilm consisting of a complex microbial community rather than a single pathogen, information on the composition of indigenous oral microbiota is important. Although the oral microbiota of several mouse strains have been characterized [2-4], these studies were based on cultivation. In addition, the isolates were identified by phenotypic characterization, including Gram staining, the catalase reaction, and commercial biochemical tests such as API strips.

It is now generally accepted that microbial community analysis should be culture-independent and utilize molecular identification methods such as sequencing of 16S rRNA genes. The typical procedure for culture-independent dissection of a bacterial community's structure involves the isolation of whole bacterial community DNA, amplification of 16S rRNA genes, cloning into an *Escherichia coli *host, and sequencing of each cloned amplicon. Recently, pyrosequencing, a new high-throughput DNA sequencing technique, has been introduced and employed in various microbiological disciplines. Pyrosequencing allows over 100-fold higher throughput than the conventional Sanger sequencing method. The higher throughput makes it possible to process large numbers of samples simultaneously and also makes it possible to detect rare species [5]. The utility of pyrosequencing in the characterization of microbial communities has been well documented for the Roche/454 Genome Sequencer (GS) 20 machine [5,6] and the GS FLX system [7-9], which produce sequence reads of approximately 100 bp and 250 bp in length, respectively. At the end of 2008, a new pyrosequencer called GS FLX Titanium was developed; it generates fivefold more sequencing reads and an extended read length (~450 bp) compared to the GS FLX system. This latest model pyrosequencer has been used for genome sequencing but has not been tested for culture-independent microbial community analysis based on 16S rRNA.

The composition of indigenous microbiota seems to be the result of strong host selection and co-evolution [10]. The role of the immune system in the selection of indigenous microbiota has been demonstrated in several studies. The total cultivable oral microbiota of athymic *nu*/*nu *mice was dominated by *Enterococcus faecalis*, while that of *nu*/+ mice was dominated by *Lactobacillus murinus *[11]. In contrast, B-cell-deficiency had no apparent influence on the indigenous oral microbiota of mice [12]. Toll-like receptors (TLRs) are innate immune receptors that recognize microbial molecular patterns and mediate innate immune responses to microbes. TLR2 recognizes the bacterial lipoproteins, lipoteichoic acids, and lipopolysaccharides of some bacterial species, including *P. gingivalis *[13]. TLR2-deficient mice clear *P. gingivalis *infection far more rapidly than control mice and resist alveolar bone loss induced by *P. gingivalis *[14]. However, it is not known if TLR2 deficiency affects the composition of indigenous oral microbiota and the colonization of *P. gingivalis*. To evaluate the effect of TLR2 deficiency on oral microbiota, oral bacterial communities of wild-type (n = 4) and TLR2 knock-out (n = 4) C57BL/6 mice were characterized using a Roche/454 GS FLX Titanium pyrosequencer. To our knowledge, this study presents the first report of a 16S rRNA-based survey of a microbial community using the Roche/454 GS FLX Titanium system with > 400 bp sequence reads.

## Results and discussion

### Collected data

We obtained a total of 102,976 reads (> 100 bp) with an average length of 449 bp from the pyrosequencing of PCR amplicons. Apparently, the Roche/454 GS FLX Titanium system produced data sets with a longer average length than those generated by earlier models (i.e., the GS20 and GS FLX systems). Barcodes embedded in both forward and reverse primers allowed sequencing of multiple DNA samples in a single run. In this study, we sequenced eight samples; however, this method could be extended to the multiplexing of hundreds of different samples using 8-bp long barcodes.

After the low quality reads and primer sequences were discarded, the final dataset contained 80,046 reads with an average length of 443 bp (excluding the PCR primer sequences). These results corresponded to 8,590 to 12,746 reads per mouse (Table 1). Non-specific short PCR products accounted for a substantial portion of the low quality reads, and gel purification of the PCR amplicons would have increased the number of passed reads. Since we only included reads that were longer than 300 bp in the final dataset, all analyzed sequences contained at least two of the V1, V2, and V3 regions [15].

**Table 1:** Data summary and diversity estimates

		WT1	WT2	WT3	WT4	KO1	KO2	KO3	KO4
Mouse age (wk)		15	11	14	15	9	9	16	16
Housing period (wk)^a^		9	3	8	9	9	9	16	16
Total reads^b^		13054	10264	13187	11625	15745	15348	11573	12180
Number of reads analyzed^c^		9840	9029	9669	8590	12746	11687	8928	9557
Average length (bp)		436	466	437	432	463	432	436	437
Maximum length (bp)		525	530	512	526	527	524	518	518
Number of phylotypes									
observed		82	162	85	87	326	106	140	108
Chao1 estimation		136	194	118	114	470	146	250	144

a Period that mice were housed at the Laboratory Animal Facility of the School of Dentistry, Seoul National University

b ≥ 100

c ≥ 300 and N = 0 or 1

### Microbial diversity in murine oral microbiota

**Figure 1:**
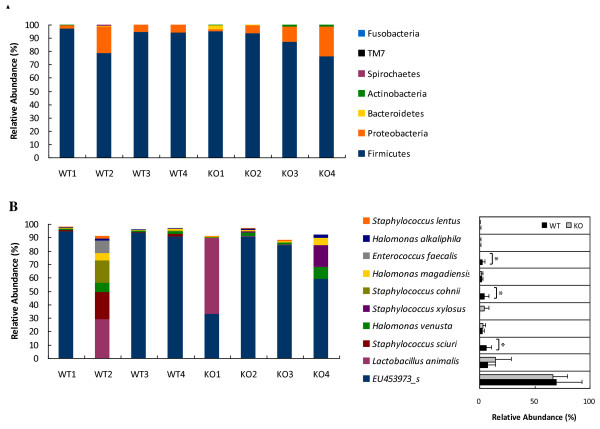
**The major phyla and species/phylotypes identified in murine oral bacterial communities**. (A) Only phyla with a mean relative abundance greater than 0.01% are shown. (B) The top ten dominant species/phylotypes are shown. The right panel presents the mean values of the WT and KO groups. *, *p *< 0.05.

Each refined pyrosequencing read was first taxonomically assigned by aligning it to the sequences in the EzTaxon-extended database, which is a new 16S rRNA sequence database that has a complete taxonomic hierarchy for the correct assignment of each sequence read. Using this new system, 97.7% of all analyzed sequences were successfully assigned from the species up to the phylum level. About 0.03% of all sequences could not be defined at the phylum level, while the rest belonged to 12 phyla. Among these 12 phyla, *Firmicutes *and *Proteobacteria *(most were from the class *Gammaproteobacteria*) encompassed the majority of sequences (> 99%). The other phyla comprised a minor portion in each mouse (Figure 1A). For the phyla *Cyanobacteria*, *Verrucomicrobia*, *Tenericutes*, *Acidobacteria *and *Planctomycetes*, less than five sequences were found in the total analyzed reads. Surprisingly, the oral microbiota from captive mice were dominated by only a few thriving species/phylotypes. Most of the phylotypes (defined by 97% sequence similarity) identified in this study were present at very low levels. The ten most frequently found species/phylotypes represented more than 88% of the oral microbiota in each animal (Figure 1B). In particular, *Streptococcus *EU453973_s, which is a tentative species (phylotype) represented by the GenBank accession no. EU453973, was the most dominant phylotype in six out of eight mice examined, and represented 59% to 94% of all sequence reads analyzed in each animal. In mouse WT2, *Streptococcus *EU453973_s accounted for only 0.02% of the total bacteria, and instead of *Streptococcus *EU453973_s, lactobacilli and staphylococci were the dominant bacteria. This finding agrees with the findings of a previous report on the indigenous cultivable oral bacteria of C57BL/6 mice [4]. An unidentified *Streptococcus *species has been previously reported to eventually dominate the murine oral microbiota by displacing the other bacterial species. This bacterium was present in mice originating from the Jackson Laboratory, but not in mice from Charles River [16]. The C57BL/6 wild-type mice used in this study were purchased from the Orient Co., which originated from Charles River. It is not possible to confirm whether the streptococci observed in the study conducted by Marcotte *et al*. [16] corresponds to *Streptococcus *EU453973_s identified in the present study, due to a lack of sequence data from the previous study. Mouse WT2 was housed at the Laboratory Animal Facility of our school for only three weeks, whereas the three other wild-type mice were housed for eight or nine weeks in the same room with the TLR2-deficient mice. Thus, the microbial community of WT2 may represent that of the mice from Charles River without the dominant *Streptococcus *species. The effect of the housing environment and the suppliers on the composition of mouse oral microbiota has been previously reported [16,17].

**Figure 2:**
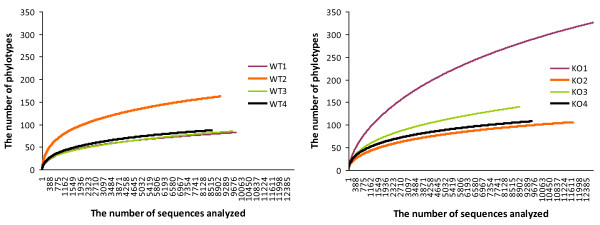
**Rarefaction analysis performed by the RDP pipeline**. Repeated samples of phylotype subsets were used to evaluate whether further sampling would likely identify additional taxa.

To determine whether all phylotypes present in the bacterial community were detected in this study, rarefaction analyses were performed. When a phylotype was defined using a threshold of 97% nucleotide sequence similarity, 82 to 326 (average 137) phylotypes were found in each mouse (Table 1). Although the gradients of collector's curves decreased quickly at approximately 1000 sampled sequences, the number of phylotypes was on the increase even at the highest numbers of sequences sampled (Figure 2). The Chao1 estimator of species richness in eight mice ranged from 114 to 470 (average 197), representing about 40% higher numbers than those observed in the present study (Table 1). Due to the known sequencing error of the Roche/454 technology and the possibility of chimeras, it is fair to say that the numbers of phylotypes calculated in this study are overestimates [18]. Trudel *et al*. [3] identified only 18 species among 671 cultivated bacterial isolates from the oral cavity of BALB/c mice. By applying the averaged rarefaction curves of our data sets, 671 sampled sequence reads would correspond to 44 phylotypes. Although the genetic backgrounds of the mice used in these two studies are different, the species diversity of murine oral microbiota determined by the culture-dependent method is only 41% of that determined by the culture-independent method. Similarly, over 60% of the 141 predominant species detected in the human oral cavity have not been cultivated [19].

Interestingly, the estimated species richness of murine oral bacterial flora is far lower than that of humans reported by Keijser *et al*. [6]. A direct comparison between the Keijser *et al*. findings and our results is inappropriate because the human data represented pooled samples from 71 individuals and was based on very short sequence reads (~100 bp). Nevertheless, the relatively low species richness of murine oral microbiota is expected due to the dominance of a small number of bacterial species.

### A comparison of oral microbiota from wild-type and TLR2-deficient mice

To evaluate the effect of TLR2 deficiency on oral microbiota, the relative abundance of each taxon at the different taxonomic ranks ranging from phylum to species was compared between wild-type and TLR2-deficient animals. The present study has limitation in that the wild-type and TLR2-deficient animals were not subjected to the same environmental conditions during the entire period. Nevertheless, a significant difference in the relative abundance was found at the species level for three species of bacteria: *Staphylococcus sciuri*, *Staphylococcus xylosus*, and *Enterococcus faecalis *(*p *< 0.05 for all three species, Figure 1B). The diversity of oral microbiota showed a tendency to increase in TLR2-deficient mice, but this finding was not statistically significant (Table 1). Collectively, the lack of the TLR2 protein had a negligible effect on the murine oral bacterial flora. Thus, the innate immune response through TLR2 seems to be dispensable for maintaining normal oral bacterial flora in mice. Wen *et al*. [20] reported that MyD88 deficiency in NOD mice changed the composition of intestinal microbiota and protected the animals from the development of type 1 diabetes, but neither TLR2 nor TLR4 deficiency protected the animals from the disease. The MyD88 protein is an adaptor protein used by multiple TLRs including TLR2 and TLR4. Although the intestinal microbiota of TLR2- or TLR4-deficient mice was not analyzed in the previous study, it is likely that a single TLR gene deficiency may not be sufficient to affect the intestinal microbiota, as TLR2 deficiency hardly affected oral microbiota.

We observed remarkably similar oral microbial communities in six out of eight animals regardless of their TLR2 genotype (Figure 1B). This is quite different from human oral microbiota, where significant inter-individual variability has been recognized [19,21]. The low inter-animal variability in murine oral microbiota may be attributed to their inbred genetic background, controlled diet, and specific pathogen-free housing conditions.

### A comparison of mouse and human oral microbiota

We successfully analyzed previously published human saliva and plaque samples [6] using our new bioinformatic system for taxonomic assignment. Clearly, the human oral microbial communities were more complex than those of the mouse, and the top ten bacterial species/phylotypes represented less than 50% of the oral microbiota in the human samples (Additional file 1). Only 27 species of identified oral bacteria were found to be shared between mice and humans (Table 2). In particular, mouse WT2 contained as many as 19 out of the 27 bacterial species, although the frequencies of these species were substantially different from those observed in humans. In the other animals, only three to five common bacterial species were identified. These results indicate that the composition of the murine oral microbiota is significantly different from that of humans, which may partly explain why mice do not develop periodontitis. Although *P. gingivalis*-induced periodontitis has served as an animal model for periodontitis [1], *P. gingivalis *(or other species in the genera *Porphyromonas*) was not part of the normal murine oral flora. Interestingly, the 19 bacterial species shared between mouse WT2 and the humans included *Fusobacterium nucleatum *and *Treponema denticola*, which are known to be associated with periodontitis [22]. Whether or not the presence of these human-associated bacteria in the mouse oral cavity affects the colonization of *P. gingivalis *and susceptibility to *P. gingivalis*-induced periodontitis warrants further investigation.

**Table 2:** Bacterial species shared between mouse and human oral microbiota

		**Mouse**^a^								**Human**^b^	
Species		WT1	WT2	WT3	WT4	KO1	KO2	KO3	KO4	Saliva	Plaque
*Actinomyces massiliensis*			0.02							0.014	0.905
*Actinomyces naeslundii*^c^			0.02							-	-
*Brevundimonas diminuta*^c^			0.01							-	-
*Corynebacterium accolens*						0.01				0.003	0.002
*Corynebacterium durum*			0.01							0.152	0.775
*Corynebacterium matruchotii*			0.07							0.192	8.934
*Corynebacterium tuberculostearicum*						0.01				0	0.009
*Enterobacter cancerogenus*^c^									0.01	-	-
*Enterococcus faecalis*^c^		0.04	9.04	0.02	0.01					-	-
*Fusobacterium nucleatum*			0.02		0.07					0.824	3.219
*Gemella haemolysans*^c^			0.01							-	-
*Haemophilus parainfluenzae*			0.03							3.761	3.110
*Kingella denitrificans*							0.01			0.103	0.304
*Lactobacillus johnsonii*								0.01		0.001	
*Neisseria subflava*			0.01							4.420	0.051
*Propionibacterium acnes*		0.21	0.03	0.01	0.02		0.03	0.95	1.21	0.017	0.150
*Rothia aeria*			0.02							0.208	1.048
*Staphylococcus hominis*							0.02				0.002
*Staphylococcus saprophyticus*									0.01		
*Staphylococcus sciuri*		1.36	20.32	0.56	1.66	0.03	0.01			0.001	0.003
*Streptococcus mitis*^c^			0.01					0.01		-	-
*Streptococcus pseudopneumoniae*		0.03								4.890	2.344
*Streptococcus salivarius*			0.02		0.02					3.747	0.029
*Streptococcus sanguinis*			0.12							11.145	9.028
*Treponema denticola*^c^		0.03	0.72				0.03		0.01	-	-
*Triticum aestivum*									0.02	0.001	
*Veillonella parvula*			0.01							0.003	
SUM^d^		1.88	30.74	0.67	2.44	0.04	0.12	1.20	1.50	32.942	29.935

^a^The relative abundance (%) of bacterial species observed in this study. Bacterial samples from the tongue, palate, and incisors were pooled.

^b^The relative abundance (%) of bacterial species obtained from an analysis of data generated by Keijer et al. [6]. Saliva from 71 individuals and supragingival plaque from 98 individuals was pooled.

^c^Not present in the study by Keijer *et al*. but found in the study by Paster *et al*. [24]

^d^Total contribution of bacterial species shared between mouse and humans

## Conclusion

To our knowledge, this study presents the first successful application of the Roche/454 FLX Titanium to 16S rRNA-based microbial community analysis. Using this new method, the oral bacterial community of captive mice was found to be relatively simple, consisting mainly of a few species in the genera *Streptococcus*, *Staphylococcus*, *Lactobacillus*, *Halomonas *and *Enterococcus*. In addition, the mouse oral bacterial community was not affected by TLR2 deficiency. This survey provides a basis for future studies of the role of periodontal pathogens in the murine model of periodontitis.

## Methods

### Mice

TLR2-deficient mice of the C57BL/6 background were kindly provided by Shizuo Akira (Osaka University, Japan) and have been bred and maintained at the Laboratory Animal Facility of our school in pathogen-free conditions for five years. Pathogen-free wild-type (WT) C57BL/6NCrljBgi mice were 6 or 8 weeks old upon purchase from the Orient Co. (Kyung-gi, Korea) and were housed on the same rack with the TLR2-deficient mice for 3 to 9 weeks to exclude the effect of environmental factors on oral microbiota. The diet used at the Laboratory Animal Facility of our school and at the Orient Corporation was the same: irradiated Rodent Diet 20 (Orient) and filtered sterile water. All of the mice were male. The handling of the animals and experimental protocols were approved by the Seoul National University Animal Care and Use Committee.

### Bacterial DNA extraction from oral tissues

Pieces of tongue, palate, and incisors (including the periodontium) were excised and subjected to bacterial genomic DNA (gDNA) extraction using a commercial kit (iNtRON, Kyung-gi, Korea). Briefly, the tissues were treated with lysozyme at 37°C for 15 min and lysed with a buffer containing proteinase K and RNase A at 65°C for 15 min. Subsequently, the lysates were mixed with binding buffer and the gDNA was purified using resin columns.

### Amplification of 16S rRNA gene and sequencing

The extracted gDNA was amplified using primers targeting the V1 to V3 hypervariable regions of the bacterial 16S rRNA gene (V1-9F: 5'-*X*-AC-GAGTTTGATCMTGGCTCAG-3' and V3-541R: 5'-*X*-AC-WTTACCGCGGCTGCTGG-3' where *X *denotes an 8 nucleotide long barcode uniquely designed for each mouse followed by a common linker AC). In this study, fixed length barcodes were used. However, enhanced sequencing results were obtained using mixtures of barcodes with varied lengths (6 to 10 bp). PCR reactions were carried out in a thermocycler (MJ Research, Reno, USA) under the following conditions: initial denaturation at 94°C for 5 min; followed by 25 cycles of denaturation at 94°C for 30 sec, annealing at 60°C for 30 sec, and elongation at 72°C for 1 min 20 sec. The amplified products were purified using resin columns, and 1 μg of PCR product for each mouse was mixed and subjected to pyrosequencing. The DNA sequencing was performed by Macrogen Incorporation (Seoul, Korea) using the standard shotgun sequencing reagents and a 454 GS FLX Titanium Sequencing System (Roche), according to the manufacturer's instructions.

### Pre-processing of data sets

Sequencing reads from the different samples were separated by unique barcodes. Then, barcode, linker, and PCR primer sequences at both sides were removed from the original sequencing reads. The resultant sequences were subjected to a filtering process where only reads containing 0-1 ambiguous base calls (Ns) and 300 or more base pairs were selected for the final bioinformatic analyses. Non-specific PCR amplicons that showed no match with the 16S rRNA gene database upon BLASTN search (expectation value of > 10^-5^) were also removed from the subsequent analyses. The pyrosequencing data are available in the EMBL SRA database under the accession number ERA005744.

### Taxonomic assignment of individual sequencing reads

For taxonomic assignment of each pyrosequencing read, we used an extension of the EzTaxon database http://www.eztaxon.org[23], which stores 16S rRNA gene sequences of type strains of validly published names. In addition to the sequences of type strains, this newly developed database, designated as EzTaxon-extended database http://www.eztaxon-e.org, contains representative phylotypes of either cultured or uncultured entries in the GenBank public database with complete hierarchical taxonomic classification from phylum to species. Representative phylotypes were designated as tentative species with artificially given specific epithets. For example, the specific epithet *Streptococcus *EU453973_s was given for the GenBank sequence entry EU453973, which plays a role as the type strain of a tentative species belonging to the genus *Streptococcus*. Similarly, tentative names for taxonomic ranks that were higher than species were also assigned where appropriate. Using this approach, the presence of species that have not yet been described can be compared across multiple bacterial community datasets. Details of the EzTaxon-extended database and software for related bioinformatic analyses will be published elsewhere.

Each pyrosequencing read was taxonomically assigned by comparing it with sequences in the database using a combination of initial BLASTN-based searches and pairwise similarity comparisons as described by Chun *et al*. [23]. We used the following criteria for taxonomic assignment of each read (*x *= similarity): species (*x *≥ 97%), genus (97 > *x *≥ 94%), family (94 > *x *≥ 90%), order (90 > *x *≥ 85%), class (85 > *x *≥ 80%), and phylum (80 > *x *≥ 75%). If the similarity was below the cutoff point, the read was assigned to an "unclassified" group. Previously published pyrosequencing data for human saliva and plaque bacterial communities [6] were obtained from the public domain and also processed using the same bioinformatic pipeline based on the JAVA programming language.

### Calculation of species richness and diversity indices

The diversity, species richness indices, and rarefaction curves were calculated using the Ribosomal RNA database project's pyrosequencing pipeline http://pyro.cme.msu.edu/. The cutoff value for assigning a sequence to the same group (phylotype) was equal to or greater than 97% similarity.

### Statistics

The differences between WT and TLR2-deficient mice were analyzed with the Mann-Whitney U-test using SAS 9.1.3 software. The statistical significance was set at *p *< 0.05.

## List of abbreviations

TLR: Toll-like receptor; WT: wild-type; KO: knock-out.

## Competing interests

The authors declare that they have no competing interests.

## Authors' contributions

JC designed bioinformatics, analyzed and interpreted results, and wrote the manuscript. KYK sampled the bacterial gDNA and prepared PCR samples for pyrosequencing. JHL participated in bioinformatic analyses. YC designed the studies, interpreted results, and wrote the manuscript. All authors read and approved the final manuscript.

## Supplementary Material

Additional file 1 **Relative abundance of the major phyla and species/phylotypes identified in human oral bacterial communities**. The previously published data of human plaque and saliva were analyzed using a new bioinformatic system for taxonomic assignment. The relative abundance of phyla (A) and top 10 species/phylotypes (B) are shown. Click here for file
