# Supplementary figures and images for: The analysis of oral microbial communities of wild-type and toll-like receptor 2-deficient mice using a 454 GS FLX Titanium pyrosequencer
Source: BMC Microbiol. 2010 Apr 6;10:101. doi: 10.1186/1471-2180-10-101 (PMC2873484; doi:10.1186/1471-2180-10-101)

## Slide 1
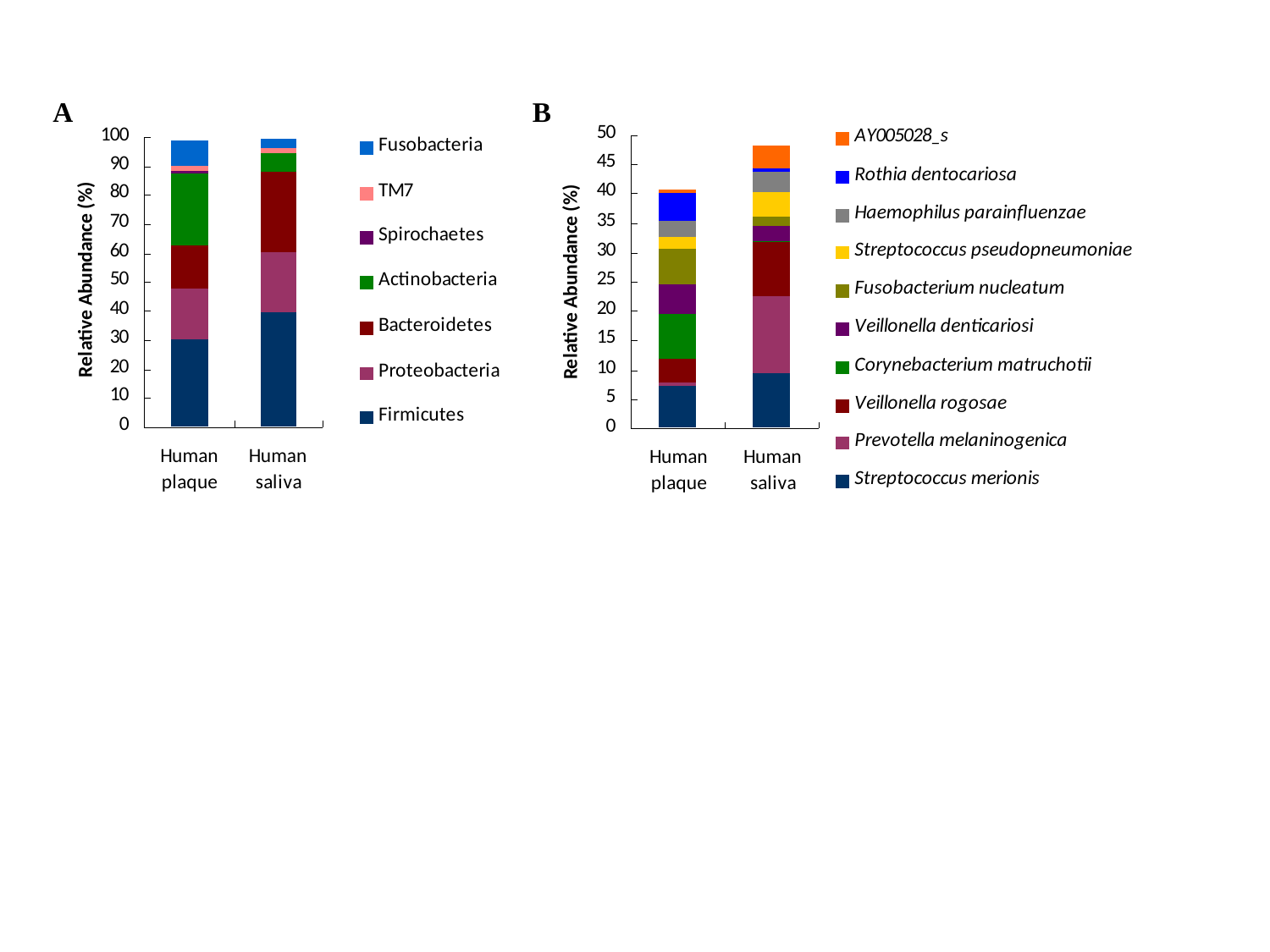

A
B

Supplement: Additional file 1 — Relative abundance of the major phyla and species/phylotypes identified in human oral bacterial communities. The previously published data of human plaque and saliva were analyzed using a new bioinformatic system for taxonomic assignment. The relative abundance of phyla (A) and top 10 species/phylotypes (B) are shown. [file 1471-2180-10-101-S1.PPT]
